# Estimating the Demand for a Preventive HIV Vaccine: Why We Need to Do Better: Reliable estimates would help in achieving several policy and advocacy objectives

**DOI:** 10.1371/journal.pmed.0030398

**Published:** 2006-09-05

**Authors:** Robert Hecht, Chutima Suraratdecha

## Abstract

Additional research on public and private demand for HIV vaccines is needed to strengthen ongoing advocacy and planning for eventual vaccine introduction, say Hecht and Suraratdecha.

At the end of 2005, about 40 million people worldwide were living with HIV/AIDS, most of them adults and 95 percent of them living in developing countries. Although vigorous prevention programs are under way in many countries, about 3 million people still died from AIDS in 2005 and more than 13,000 people became infected with HIV every day [1]. Hence there is an urgent need for stronger and more effective HIV prevention. New technologies are required to augment existing prevention methods. An HIV vaccine could make an enormous difference in the effort to reverse the pandemic, thereby saving tens of millions of lives.

Vaccines have been highly cost-effective in fighting other infectious diseases such as polio, smallpox, hepatitis B, yellow fever, and a range of childhood illnesses. A safe, effective, and accessible preventive HIV vaccine would be a hugely valuable addition to the existing array of prevention measures. In a number of modeling exercises, analysts have shown that even a partially effective vaccine could decisively lower the rate of new infections, averting millions of new infections each year [2–4].

In the global effort to develop an HIV vaccine, more than 30 vaccine candidates are currently in trials in 19 developed and developing countries [5]. Among these, a large-scale phase III trial is under way in Thailand, and a major phase IIb trial involving several thousand volunteers is ongoing in Latin America, the Caribbean, Europe, and North America to obtain proof of concept for a vaccine built on an adenovirus vector platform [6]. The results of these trials over the next few years will help us set a more definitive timeline for the eventual launch and introduction of a vaccine.

## Why Estimates of Demand Matter

For a vaccine to make a difference in halting the AIDS pandemic, it needs to be widely available and accessible, and must be taken up. The vaccine must be demanded by individuals and government authorities. Reliable estimates of demand for an HIV vaccine would help in achieving several policy and advocacy objectives related to this goal, including: (1) persuading governments and industry to invest more in research and product development; (2) convincing donors to finance vaccine purchases, in advance or at the time of uptake, given the limited ability of governments and individuals in poor developing countries to pay for a vaccine; (3) helping industry to determine the scale of manufacturing facilities to build to ensure adequate production capacity; (4) guiding research portfolio management decisions by highlighting how vaccine characteristics may affect demand; and (5) guiding governments in planning their HIV vaccination programs, including developing information campaigns and strengthening vaccine delivery systems.

A successful HIV vaccine is likely to have characteristics that make estimating demand especially challenging. Unlike most existing vaccines that are aimed at children, an HIV vaccine will probably be recommended for adolescents and adults. From a public health perspective, an HIV vaccine is likely to have the largest epidemiological impact when targeted at groups with high risk of infection, such as commercial sex workers (CSWs) and injecting drug users (IDUs). However, little is known about the uptake of vaccines and other health services by adults and marginalized social groups in developing countries. The first generation of licensed HIV vaccines may also be only partially effective in protecting against infection and at the same time very expensive, which could reduce the vaccine's acceptability to both governments and individuals. Furthermore, the stigma associated with HIV/AIDS, if not adequately addressed, may lower demand for a vaccine.

For these reasons, ascertaining the private and public demand for an HIV vaccine is important—but until now, such estimates have been incomplete and based on product profiles (efficacy, mode of action, duration) that differ substantially from the profiles of the HIV vaccines currently in the research and development pipeline.

It is therefore critical that a renewed effort be made to develop and implement models for HIV vaccine demand that capture the key drivers of expected demand; are based on the latest and best scientific knowledge; and are flexible and can be updated periodically as the vaccine field advances in the coming years.

## What Do We Know about HIV Vaccine Demand?

To assess the state of HIV vaccine demand analysis and the need for further work in these areas, a team from the International AIDS Vaccine Initiative (IAVI) reviewed the existing scientific literature, including 18 previous studies (see bibliography available at http://www.iavi.org/annotated). The results from these studies were synthesized [7].

### Global estimates

At the global level, three estimates of demand for an HIV vaccine have been made to date. The first focuses on the number of vaccination courses required by developing countries for national HIV vaccination programs in the first five years after vaccine launch [8]. (A “course” refers to two or more vaccinations administered to the same individual several months apart, as is done for many vaccines currently in use.) The second used marginal benefit and opportunity cost analysis to estimate the volume of vaccine purchases globally [9]. The most recent study is based on public health needs and probable vaccine uptake in developed and developing countries [10].

Results of these studies are presented in Figure 1. The three studies converge on an estimated demand for the developing world of several hundred million vaccine courses in the early “catch-up” years when large numbers of adults are being vaccinated. Because of constraints on accessibility and acceptability, one study argues that effective uptake of an HIV vaccine would only be 49 million courses for a low- to moderate-efficacy vaccine, but would reach 260 million courses for a high-efficacy product in the first five years after its introduction.

**Figure 1:**
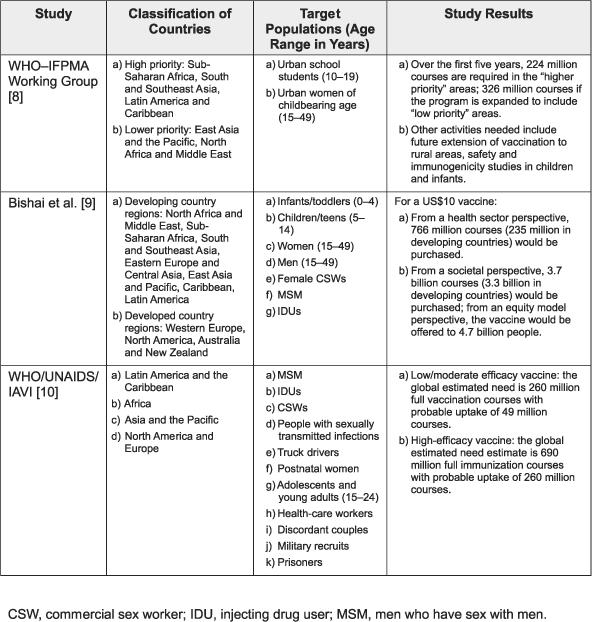
Results from Global Public Sector Demand Studies

### Country-level estimates

Four studies have looked at public sector demand for an HIV vaccine at country level for Thailand, Brazil, Southern Africa, and Southern India [11–14]. The population groups assumed to be vaccinated in each study varied in response to local political and epidemiological factors. In the Southern India, Southern Africa, and Thailand analyses, groups were prioritized for vaccination on the basis of cost-effectiveness. Relative costs were adjusted to reflect the difficulty of reaching those population groups. In calculating the number of infections averted, the Thailand and Brazil studies considered whether an HIV vaccine would complement or substitute for existing HIV prevention methods such as condoms. The Brazil study did not use cost-effectiveness because the authors believed that the prevailing national political outlook on AIDS and health would require the government to vaccinate every individual, regardless of risk status.

The studies made different assumptions about the share of population groups vaccinated—partial or complete coverage—at the time of product introduction. The study of Southern Africa argues for targeting high-risk groups and for providing vaccination to individuals after they receive voluntary counseling and testing. The Southern India study developed vaccine delivery scenarios for each population group. The Thailand study proposes that vaccines be allocated strictly on the basis of relative cost-effectiveness across groups, for given levels of government budget resources.

A simple vaccine product profile was used in all four studies, typically a single dose with 100 percent efficacy. For Southern Africa and Southern India, it was assumed that the vaccine would only offer protection from HIV infection for five years, while the Brazil and Thailand studies assumed lifetime protection. A series of prices from US$1 to US$10 a dose was considered, except in Thailand where the price ranged up to US$28 per dose.

The potential demand was calculated by multiplying population size by the estimated coverage rate (Figure 2). The required budget was then estimated by multiplying price by the number of doses.

**Figure 2:**
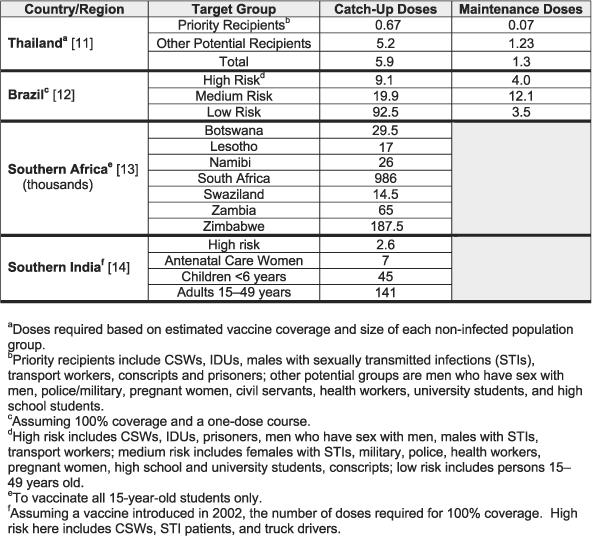
Number of Doses of HIV Vaccine Required by the Public Sector (in Millions, Unless Otherwise Indicated), Four Country Studies

The four studies suggest that the number of doses of HIV vaccines needed in the initial phase could be large if broad targeting is followed—over 121 million doses in Brazil and 195 million doses in Southern India. If a narrower targeting strategy to reach only high-risk populations were followed, however, the number of doses needed would be significantly smaller—9.1 million doses in Brazil for those at greatest risk, 9.6 million doses in Southern India when focusing exclusively on high-risk individuals and women receiving antenatal care services, and 1.3 million doses for 15-year-old school students in Southern Africa. The Thai study argues that 5.9 million doses would cover priority groups in the country, with an additional 1.3 million doses needed annually to maintain coverage.

### Private demand

Studies have been conducted in four countries—Kenya, Mexico, Thailand, and Uganda—among samples of the adult population, such as patrons of shopping centers, households in various communities, students, and adolescents [15–19]. One study included respondents at high risk of infection: CSWs and IDUs [15].

Efficacy level, number of doses, duration, and price were the most commonly used vaccine characteristics. Although most studies explored demand for a partial-efficacy vaccine, the Thailand study was the first to develop a tool to explain this concept to respondents and check their understanding [16].

Figure 3 reports the proportion of individuals who expressed willingness to pay (WTP) for an HIV vaccine. Even though less than 100 percent of population groups studied might benefit from being vaccinated, the reported level of demand is still high, even when individuals were asked to pay out of pocket. In Uganda, more than three-quarters of the general population said they would purchase a vaccine at about US$3 a dose, while in Thailand more than three-quarters of CSWs and IDUs would buy a vaccine at US$25 a dose.

**Figure 3:**
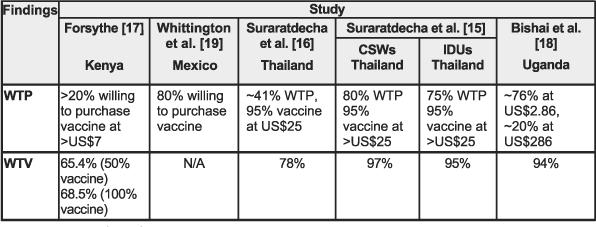
Reported Willingness to Pay and to Be Vaccinated in Four Countries

When asked about an HIV vaccine offered free of charge, the proportion of respondents who expressed willingness to be vaccinated (WTV) increased to 78 percent and 94 percent of the general population in Thailand and Uganda, respectively, and 97 percent of CSWs and 95 percent of IDUs in Thailand (Figure 3). In Kenya, where vaccine price was not explored, WTV was about 65 percent for a 50 percent effective vaccine and slightly higher for a 100 percent effective vaccine.

Regression analysis found that the general population in the two East African countries and respondents at high risk for HIV infection in Thailand showed indifference to vaccine efficacy [15–18]. In contrast, the sample from the general population in Thailand, facing a longstanding but less severe epidemic, showed a preference for a higher-efficacy vaccine at any given price [16]. This confirms the role of perceived HIV risk in determining the demand for an HIV vaccine.

The study in Thailand explored the comparative proportions of respondents in each of three groups—the general population, CSWs, and IDUs—willing to pay for an HIV vaccine at prices from US$12.50 to US$500 [15]. CSWs were the most likely to pay at a given price, followed by IDUs and the general population. Willingness to pay fell for all groups as price rose, dropping below 25 percent at the US$500 level for the general population even for a 95 percent effective vaccine.

Two of five studies explored the effect of vaccine price and found that higher prices reduce demand [16,18]. They also suggest that greater understanding of vaccine efficacy can raise demand and willingness to pay for a vaccine. This is important for the design of future promotional campaigns to persuade people to participate in HIV vaccination.

### Lessons for policy makers

The existing literature on demand for HIV vaccines points to several important lessons for policy makers. First, with a potential demand of several hundred million doses in developing countries during an initial period, HIV vaccine manufacturers should be persuaded that this represents an attractive market in volume terms, with the price per dose as the main uncertainty. To address this latter issue, developed country governments could guarantee a reasonable price (e.g., US$5–US$10 per dose) for an HIV vaccine that met certain technical performance standards. A pilot scheme along these lines is being recommended to the G8 governments under the current advance market commitment proposal for new vaccines (http://www.cgdev.org/section/initiatives/_active/vaccinedevelopment).

Second, since the vaccine delivery and targeting strategies followed by developing countries will significantly affect the size of global demand, it is imperative that market forecasts for an HIV vaccine take into account these country-specific targeting preferences. These preferences are especially important in countries with large populations such as India, China, South Africa, Ethiopia, and Nigeria, since demand from these countries will drive the global demand numbers.

A third lesson for policy makers is that with private demand for an HIV vaccine among low-risk groups likely to be substantially affected by price, governments and donors will need to design polices to reduce the price of the vaccine to consumers, especially for low-income households. Direct public sector subsidies backed up by donor commitments and publicly financed health insurance schemes are two approaches that could help to make a vaccine affordable in these settings, thus transforming public health need into actual demand and uptake.

## Why Do We Need Better Estimates, and How Should They Be Done?

While suggestive and helpful, existing studies of global, regional, and country-level demand for a future HIV vaccine are inadequate and need to be significantly improved. Why?

First, they assume optimistically that all countries would adopt the vaccine in year 1, without staggering or sequencing introduction. Although many national leaders and international partners will no doubt endeavor to see that countries adopt an effective HIV vaccine as quickly as possible, past experience with other vaccines has shown that some countries are fast adopters and others are slower [20,21]. This needs to be taken into account in future uptake scenarios to make them more realistic.

The starting date for an AIDS vaccination program, the speed with which the vaccine will be taken up, the population targeting and vaccine delivery strategies, and the maximum achievable coverage level will vary from country to country, driven by a range of factors that need to be carefully explored and incorporated into demand scenarios. These include: (1) knowledge of HIV/AIDS; (2) awareness of the vaccine and its potential benefits; (3) the strength and reach of existing national and local vaccination and health programs; (4) human resources and management capacity; (5) political leadership and community mobilization capacity; and (6) cultural variables related to the stigma surrounding HIV/AIDS and AIDS prevention services generally. Data are already available for a number of these factors, and others can be estimated using proxy variables for which data also exist. In some instances, additional data will need to be collected.

Second, a number of the existing HIV vaccine demand studies do not rigorously examine the effects of vaccine price on government or individual private decisions. Furthermore, in cases where price has been considered, the price levels tested are not consistent across population groups and countries in a manner that allows for straightforward international comparisons. In the future, it would be useful to undertake additional analysis of public and private willingness to pay for an HIV vaccine, using agreed-upon standardized price levels that are consistent across countries with similar per capita incomes.

While public sector demand is extremely important to ascertain, given the dominant role of governments in sponsoring and paying for vaccines in both developed and developing countries, private demand and price sensitivity are also important, especially in middle-income countries where the private sector is often highly dynamic in the health-care field. In these settings, studies of private willingness to pay can generate important insights into strategies for government subsidies that would be equitable and help to augment overall vaccine uptake.

Third and finally, the existing vaccine demand studies use vaccine characteristics that are internally inconsistent and do not reflect current scientific opinion. Current opinion suggests that the first HIV vaccines licensed for use may be only partially effective and may act in three ways: (1) by preventing some but not all vaccinated persons from becoming infected (“partial efficacy” or “reduced susceptibility” to HIV infection); (2) by lowering a vaccinated individual's infectiousness (and thus his or her ability to infect others); and (3) by reducing the rate at which an individual would progress to AIDS disease if infected with HIV.

A standardized and up-to-date profile of these hypothetical first-generation HIV vaccines, based on the latest scientific knowledge, would help to generate the most policy-relevant and comparable results from future vaccine demand assessments.

## Conclusion

While we have already gained some valuable understanding of public and private demand for HIV vaccines from existing studies, additional research on these topics is needed to strengthen ongoing advocacy and planning for eventual vaccine introduction. This type of demand analysis can also benefit from parallel efforts to improve demand forecasting for other vaccines and pharmaceutical products and from deeper involvement of modelers, economists, and policy makers from developing countries where the AIDS epidemic is most acute.

IAVI and its partners in HIV vaccine development should seize this opportunity to mount a focused program of policy research into vaccine demand, globally and at country level. The World Health Organization, the Joint United Nations Program on HIV/AIDS, the United States National Institutes of Health, other national medical research institutions, and a number of health policy organizations all have much to offer to this effort. If combined effectively with parallel policy analysis on vaccine impact, cost-effectiveness, and strategies for financing and delivering an HIV vaccine, such additional work on vaccine demand could make an important contribution to speeding up development and access to a vaccine that could help to stop the epidemic and end AIDS.

## Abbreviations

CSW: commercial sex worker
IAVI: International AIDS Vaccine Initiative
IDU: injecting drug user
MSM: men who have sex with men
STI: sexually transmitted infection
WTP: willingness to pay
WTV: willingness to be vaccinated
